# Research on the control rate of hypertension under family physician-contracted service

**DOI:** 10.1186/s12875-024-02280-0

**Published:** 2024-01-31

**Authors:** Yiping Zheng, Yuqing Liu, Dongyu Xue, Zhao Shang, Baoquan Zhang, Yue Dai

**Affiliations:** 1 School of Health Management, Fujian Medical University, Fuzhou, 350122 China; 2 Fujian Maternity and Child Health Hospital College of Clinical Medicine for Obstetrics & Gynecology and Pediatrics, Fujian Medical University, Fuzhou, 350000 China

**Keywords:** Family physician, Hypertension, Control rate, meta-analysis

## Abstract

**Background:**

Hypertension is one of the global public health problems. Family physician-contracted service (FPCS) is widely used in the health management of hypertension patients in China. The purpose of this study was to assess the effect of FPCS on hypertension control.

**Methods:**

PubMed, Web of Science, the Cochrane Library, China National Knowledge Network, Chinese Scientific and Technological Journal Database (CQVIP), and Wanfang Database were searched for randomized controlled trials related to family physician-contracted service and hypertension control effect, and meta-analysis was performed on the literature meeting the inclusion criteria. The source of heterogeneity was discovered by meta-regression, and it was further investigated by subgroup analysis. The risk difference (RD) and 95% confidence interval (CI) were utilized as effect values. Evaluations of publication bias and sensitivity analysis were also conducted.

**Results:**

A total of 46 studies were included, and the pooled RD suggested that FPCS could effectively improve the control rate by 19% (RD = 0.19; 95%CI: 0.16–0.21; *P* < 0.001; I^2^ = 59.3%). The average age (β = 0.28; *P* = 0.05) and the intervention mode (β = 0.36; *P* < 0.001) were found to be heterogeneous sources by the meta-regression. According to subgroup analysis, the hypertension control rates of the elderly and working-age population in the experimental group were 93.6% and 90.1%, respectively; the control rates of the “family physician” mode (FP), “family physician + patient” mode (FPP) and “family physician + patient + family member” mode (FPPF) in the experimental group were 90.1%, 94.4%, and 92.6%, respectively. The sensitivity analysis revealed steady results, with no discernible publication bias.

**Conclusions:**

The FPCS is beneficial to the control of hypertension. The control effect is influenced by average age and intervention mode. The control effect of hypertension in the elderly is better than that in the working-age population, and FPP and FPPF are more beneficial to the management of hypertension than FP. The quality and continuity of FPCS should receive more focus in the future, patient self-management and family support are also essential for managing hypertension.

**Supplementary Information:**

The online version contains supplementary material available at 10.1186/s12875-024-02280-0.

## Introduction

Hypertension is the most common risk factor for cardiovascular diseases, which is usually defined as systolic blood pressure (SBP) ≥ 140mmHg or diastolic blood pressure (DBP) ≥ 90mmHg when blood pressure is measured three times on different days without antihypertensive drugs. There isn’t a fully curative treatment for it at the moment due to its very complex pathophysiology and contributing elements [1]. An estimated 1.28 billion adults globally, aged 3–79, had a diagnosis of hypertension as of 2019, accounting for roughly 32–34% of the world’s population [2]. About 330 million people in China are thought to have cardiovascular disease (CVD), of which 245 million have hypertension [3]. With the aggravation of global aging, the disease burden caused by hypertension has become a noteworthy public health problem in China and even in the international community.

In practice, the prevention and treatment of hypertension in China have achieved remarkable results in the past 20 years [4]. To address the unbalanced distribution of medical resources, and to gradually meet the needs of continuous and comprehensive medical services for hypertension patients, China began to advocate for hypertension prevention and treatment to be placed in the community around 2000 [5]. The first standard of hypertension prevention and control in primary care was issued in 2002, which promoted hypertension management technology in primary care, emphasized community-wide prevention and control, and focused on chronic disease risk factors [6]. The standardized management of hypertension has been incorporated into the national basic public health service project since the new health care reform was implemented in 2009. This means that community health service centers, township health centers, and other primary medical institutions must strictly adhere to the “National Basic Public Health Service Standard” for the management of hypertension patients within their jurisdictions [4]. Establishing paper and electronic health records, giving at least four face-to-face follow-up services each year, testing blood pressure for patients free of charge, and providing health education and lifestyle advising services for patients free of charge are all part of the management [7]. Additionally, when updates are made to the National Basic Public Health Service Standards, the standardized management will be enhanced over time.

According to a study conducted between 2012 and 2015, China’s population now has a 15% rate of hypertension under control, which is 9% higher than it was in 2002. Effective control of hypertension has been accomplished through the National Basic Public Health Service Project. However, international experience shows that only when the control rate of high blood pressure among the population is ≥ 30%, can the incidence rate and mortality of cardiovascular disease among the population be reduced [8]. Research has indicated that the relative dearth of medical resources in the community, the low quality of treatment, and some residents’ low self-care awareness all have an impact on the effectiveness of community management of chronic diseases [9, 10]. There is still a long way to go for community management of hypertension in China.

Apart from the persistently alarming epidemic situation of chronic non-communicable diseases, China’s medical system is still beset by issues like unequal resource allocation, exorbitant medical expenses, and severe service fragmentation. A “panacea” was created in the form of the FPCS. The State Council proposed that family physician services (FPCS) be the future path of health services development in China in its “Notice on Issuing the Guidance on Promoting the Signing of Family Physician Services” [11]. The exploration of Chinese-style FPCS officially kicked off. FPCS is a personalized medical service provided by family physicians after residents sign a service agreement with grassroots medical institutions for some time. This type of full-cycle, whole-population health management service is better suited for managing hypertension over the long term. It can assist contracted residents in adopting healthy lifestyles, exploring the workings of the hierarchical medical system, and gradually achieving the goal of obtaining greater health output with less health input [12].

Based on continuous practice and reference to international chronic disease management, China has gradually formed several representative FPCS models, as follows: (1) The “integrated Medical treatment and nursing care system” contracted service model in Hangzhou. The primary goals are to provide health management services that integrate medical treatment, pensions, and rehabilitation for insured people, as well as to integrate basic medical resources within the community [13]. (2) “1 + 1 + 1” contracted service model in Shanghai. Residents voluntarily select a family doctor from the community health service center, district, and municipal medical institutions to sign a contract, so that the above three levels of medical institutions can form an alliance and effectively manage patients in terms of medical treatment, referral, and medical insurance [14]. (3) The “co-management of three types of doctors” contracted service model in Xiamen. It refers to a management team comprised of a general practitioner, a health manager, and a specialty doctor who collaborate to complete the entire process of chronic disease management for patients [15]. These models do, however, still have certain common issues, such as a lack of family doctors, a shortage of resident service utilization, and inadequate information construction [16].

In light of the severe prevalence of hypertension, assessing FPCS’s efficacy is practically significant. Furthermore, the majority of studies on the impact of FPCS on the management of hypertension use experimental research using community members as case studies. These studies lack a systematic theoretical analysis framework and a thorough understanding of the variables impacting the efficiency of the services. Thus, the goal of this study is to ascertain the present state of hypertension management under FPCS and methodically assess its influencing factors.

## Methods

### Theoretical model

Dr. Anderson of the University of Chicago School of Public Health originally introduced the Behavioral Model of Health Services Use (BMHSU) in 1968, and this study is primarily based on it [17]. After five iterations and additions, the model—which was first used to examine the variables influencing family medical services utilization—has progressively grown to be regarded as a reliable framework for medical and health services research [18]. By incorporating multi-level elements impacting the rate of hypertension control into a relatively mature analysis framework, BMHSU can provide a more thorough explanation of the key traits of patients with hypertension and prevent the selection of influencing factors at random.

**Fig. 1:**
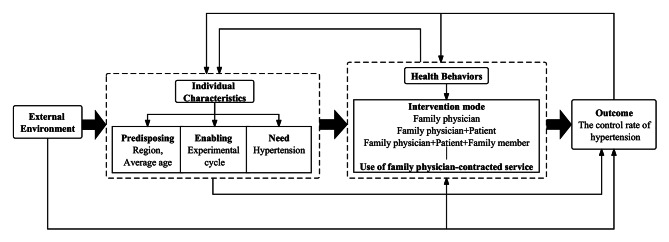
Theoretical framework of influencing factors of hypertension control rate under FPCS

As seen in Fig. 1, this paper’s theoretical framework is divided into four Sect. (1) External environment. That is, the policy environment of FPCS is confronted by a rising number of chronic patients’ objective desire for long-term health treatment. (2) Individual characteristics. This may represent the effect of specific factors on the rate at which hypertension in patients is controlled. The following three aspects are mostly examined and discussed in this article: (a) Predisposing factors. They are defined as a tendency to use health services, which reflects people’s potential to use health services. Two factors, age, and region, were included in this study; (b)Enabling factors. They correspond to the availability of FPCS, which in this study is referred to as the experimental period, representing the length of time individuals have access to FPCS; (c) Need factors. People use medical services for a variety of reasons, the most common of which are personal health requirements. In this study, hypertension is the need factor. (3) Health behaviors. Primarily consist of the health service providers’ intervention strategies and the health service demanders’ use of FPCS. Intervention mode refers to different family physician contract service models, including the “Family physician” model (FP), the “Family physician + Patient” model (FPP), and the “Family physician + Patient + Family member” model (FPPF). (4) Outcome. The hypertension control rate is the most direct manifestation of the effectiveness of FPCS.

### Search strategy

This report was carried out in accordance with the Preferred Reporting Items for Systematic Reviews and Meta-Analyses (PRISMA) guidelines (Supplementary Material 1). Literature on hypertension control rate under contracted family physician services published from the establishment of the database to January 14, 2023, were systematically searched. The search databases included PubMed, Web of Science, the Cochrane Library, China National Knowledge Infrastructure (CNKI), the Database of Chinese sci-tech periodicals (CQVIP), and the Wanfang Database. Terms and keywords used in the searches included the following: (“family physician” OR “family physician” OR “General practitioners”) AND (“hypertension” OR “high blood pressure”) AND (“control rate” OR “control ratio” OR “effective rate”) AND (“China” OR “Chinese”), the full search strategy was provided in Supplementary Material 2.

### Criteria for included and excluded studies

#### Inclusion criteria

(a) Study subjects: hypertensive patients, and basic information such as sample size, age, and place of residence are explained; (b) Intervention measures: FPCS; (c) Study type: randomized controlled trial (RCT); (d) Outcome measures: hypertension control rate.

#### Excluded criteria

(a) Study subjects: non-hypertensive patients; (b) Intervention measures are not FPCS; (c) Study type: non-randomized controlled trial; (d) There is no clear definition of outcome indicators or lack of outcome indicators; (e) The sample size, age and other basic information of the study subjects are not stated; (f) Low-quality literature.

### Literature screening and study data extraction

The pertinent information from the included literature was extracted based on the inclusion and exclusion criteria. This included the title, author, publication time, sample size, hypertension control rate, average age, gender ratio, region, experimental cycle, intervention measures, and other key information. The control rate of hypertension includes three definitions: First, $control\ rate=(valid+average)/total\times 100\%$ (valid: blood pressure was maintained at a normal level (SBP/DBP≤140/90 mmHg) for 75% or 80% of the year; average: blood pressure was maintained at a normal level (SBP/DBP ≤ 140/90 mmHg) for more than half a year; invalid: blood pressure was maintained at an abnormal level for more than half a year). Second, $control\ rate=(valid+average)/total\times 100\%$ (valid: after treatment, the patient’s blood pressure level decreased to a normal level, and the clinical symptoms of headache and dizziness disappeared; average: after treatment, the patient’s blood pressure level decreased but did not reach the normal level, the patient was accompanied by mild headache, dizziness, and other clinical symptoms; invalid: after treatment, the patient’s blood pressure level did not change significantly, and the patient’s clinical symptoms did not relieve). Third, the control rate represents blood pressure measured at the last follow-up visit at the normal level. Two reviewers conducted it independently and cross-checked it to complete the literature screening and data extraction. Disparities were reconciled through dialogue or by consulting a third party. To guarantee the accuracy of the information gathered, double entry was performed using Excel.

### Quality evaluation

Two reviewers independently evaluated the quality of the included studies through the risk-of-bias tool recommended by the Revised Cochrane risk-of-bias tool for randomized trials (RoB2). If there was no consensus, the decision was made after discussion with the third reviewer. This tool includes the following domains: (a) risk of bias arising from randomization and allocation concealment; (b) risk of bias due to participants, investigators, and outcome assessors being aware of the intervention-group assignments; (c) risk of bias due to missing data; (d) risk of bias from the measurement of the outcome; (e) risk of bias due to selective reporting. The risk of bias can be classified into three levels: “low risk of bias,” “some concerns,” and “high risk of bias” [19].

### Statistical analysis

For the meta-analysis, Review Manager (version 5.3) and STATA (version 16.0) were utilized. A forest plot was created and the effect estimate was calculated using the risk difference (RD) and the 95% confidence interval (CI). I^2^ and *P*-value are applied to describe the heterogeneity among the studies. If there is no statistical heterogeneity among the results of the studies (I^2^ < 50%, *P* > 0.05), the fixed effect model is used. If there is statistical heterogeneity (I^2^ > 50%, *P* < 0.05), the random effects model is used [20]. Meta-regression was utilized to examine heterogeneity sources, which were further investigated as subgroup variables. Egger test [21] and inverted funnel plot were used to evaluate the publication bias of studies and test the stability and reliability of the results through sensitivity analysis.

## Result

### Study screening process and results

**Fig. 2:**
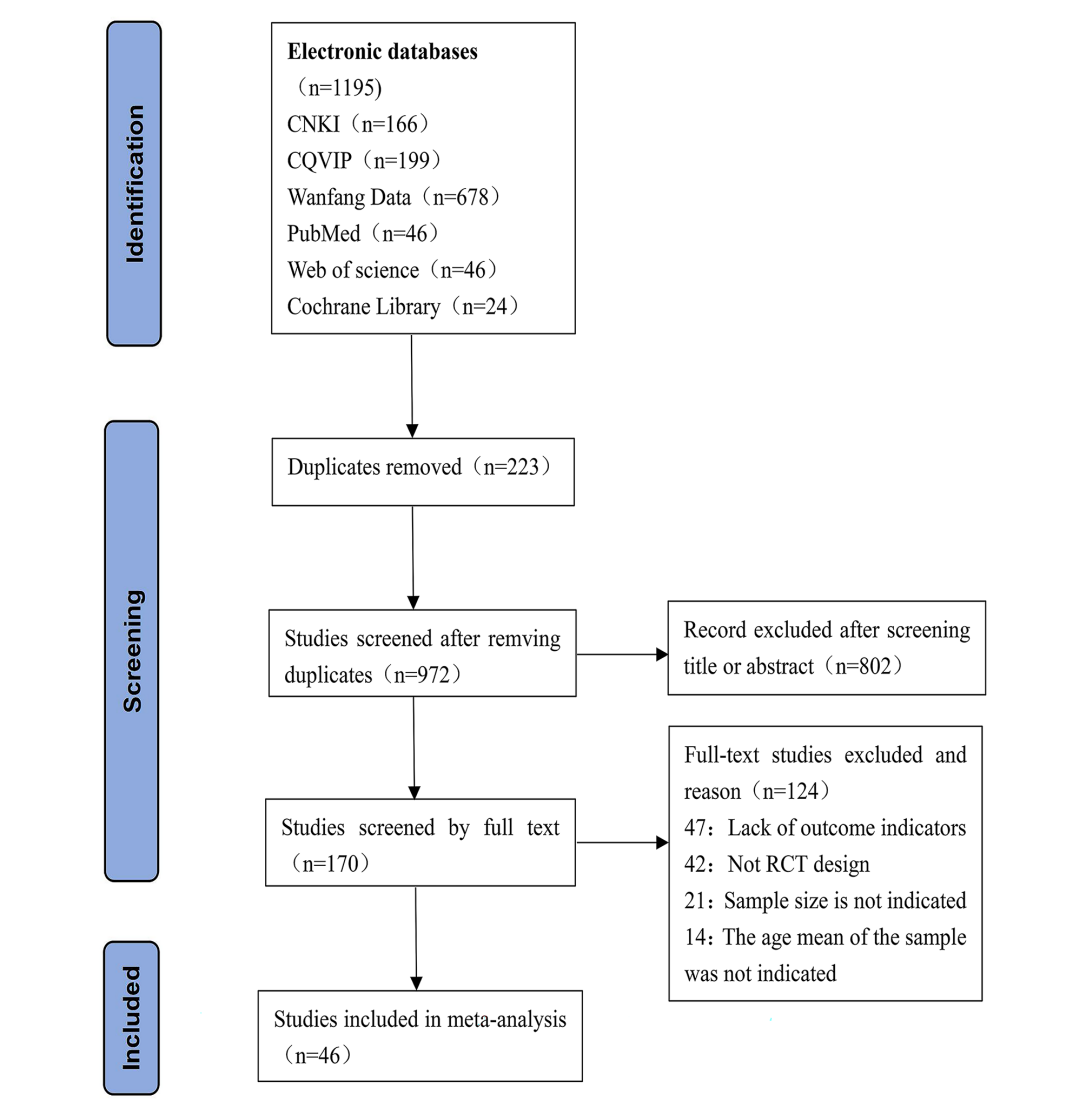
Preferred reporting items for systematic reviews and meta-analyses (PRISMA) flowchart describing literature search

According to the above literature retrieval formula, a total of 1195 relevant studies were retrieved, and then each study was excluded and confirmed one by one according to the literature inclusion and exclusion criteria. Finally, 46 studies were included, with a total sample size of 8424 people, including 4223 people in the experimental group and 4201 people in the control group (Fig. 2).

### Characteristics of studies

#### Basic information

The literature was published between 2015 and 2022, and the survey area included 17 provinces, cities, and autonomous regions in China’s east, middle, and west. There were 11 studies with an experimental cycle of less than 12 months, 21 studies with a 12-month experimental cycle, and 14 studies with a period of more than 12 months. Table 1 summarizes the basic characteristics of the included studies.

### Characteristics of intervention mode

Intervention models were classified according to the three roles involved in hypertension management. The FP denotes that the family physician is primarily responsible for patients’ health status. The intervention measures primarily consist of (1) signing a family physician service agreement; (2) establishing and maintaining health records; (3) developing personalized health intervention plans; (4) conducting follow-ups every three months or one month, including blood pressure monitoring, medication, diet, and exercise guidance; and (5) group health education, the key contents include frequent health education lectures, the distribution of hypertension prevention and control pamphlets, the formation of mutual aid groups for hypertension prevention and control. The FPP indicates that the patients will also be managed by themselves under the guidance of the family physician based on the FP. During the follow-up, family physicians used cognitive behavioral therapy, Motivational Interviewing (MI), or the Knowledge-Attitude-Practice (KAP) model to strengthen patients’ self-management abilities. The MI explores the inner contradictions of patients in the process of changing their bad behavior and finding personalized solutions through observation and interview, which is a communication method that guides patients in changing bad behavior [22]. The KAP is a knowledge, belief, and behavior intervention in which patients attain the goal of modifying behavior through three continuous processes: learning knowledge, changing attitude, and forming behavior [23]. The FPPF symbolizes the three roles involved in hypertension management. The creation of a family health worker is a feature of this model, to assist the family doctor in monitoring blood pressure, supervising medication administration, educating patients about health issues, and checking in with the patient once a week.

**Table 1:** Characteristics of the studies included in the meta-analysis

Author	Year	Control rate(%)		Experimental cycle, month	Region	Intervention mode
		Experimental group	control group			
Caixia Liu [24]	2016	87.76%	70.21%	24	east	FP
Changyue Liu [25]	2018	92.45%	71.70%	14	middle	FP
Dianbo Liu [26]	2020	94.00%	72.00%	3	east	FPP
Dongsheng Zhang [27]	2020	97.50%	87.50%	3	east	FPPF
Fudong Wang [28]	2020	100.00%	80.95%	3	west	FPP
Guicheng Li [29]	2019	91.00%	59.00%	12	east	FPPF
Haiyan Yang [30]	2022	95.83%	82.50%	29	east	FPP
Haiyan Zhu [31]	2015	91.94%	71.43%	6	east	FP
Haiyun Mo [32]	2017	90.91%	70.45%	12	east	FPP
Hong Jiang [33]	2018	95.74%	74.47%	12	east	FP
Hongyu Dou [34]	2020	96.67%	76.47%	12	east	FPP
Hui Wang [35]	2021	91.67%	70.00%	6	east	FP
Jianzhu Wang [36]	2019	81.19%	62.12%	22	east	FP
Jing Cheng [37]	2019	96.67%	80.00%	23	west	FP
Jing Dong [38]	2022	93.65%	80.95%	3	east	FP
Jiuhong Sun [39]	2022	96.67%	73.33%	12	east	FP
Lei Shi [40]	2017	92.50%	81.25%	12	middle	FP
Lihong Zhang [41]	2017	90.00%	57.00%	6	east	FPPF
Lijun Zhang [42]	2020	92.86%	71.43%	24	east	FP
Ling Tao [43]	2022	100.00%	93.10%	15	east	FPP
Ling Xue [44]	2021	97.50%	82.50%	12	middle	FP
Linlin Li [45]	2021	95.74%	80.85%	12	east	FPPF
Liting Yuan [46]	2021	97.37%	78.95%	24	east	FPP
Li Zou [47]	2107	80.00%	47.50%	12	west	FPP
Manru He [48]	2019	96.25%	82.50%	12	west	FP
Mei Yang [49]	2018	80.19%	56.60%	24	east	FP
Pei Huang [50]	2018	96.67%	80.00%	12	east	FPPF
Rongxin Zhang [51]	2019	92.00%	80.00%	6	east	FP
Shuncheng Wang [52]	2019	86.77%	55.82%	6	east	FPPF
Wei Wang [53]	2021	92.50%	70.00%	12	east	FP
Wenjuan Zuo [54]	2018	91.67%	83.33%	15	east	FP
Xiaoqin Chen [55]	2020	91.67%	61.02%	12	east	FPPF
Xia Zhang [56]	2020	93.00%	80.00%	12	east	FP
Xingxiang Zhang [57]	2018	85.00%	52.50%	10	west	FP
Xiongwei Dong [58]	2019	68.52%	52.60%	12	east	FP
Xueni Huang [59]	2020	94.44%	77.78%	12	east	FPP
Yanxia Luan [60]	2020	100.00%	81.08%	23	east	FPP
Yi Zheng [61]	2020	88.89%	71.43%	12	west	FPP
Yuan Su [62]	2017	75.93%	71.43%	18	east	FP
Yu Bai [63]	2021	93.33%	77.33%	12	west	FP
Wei Li [64]	2020	95.00%	80.00%	9	middle	FP
Yue Chen [65]	2020	72.00%	55.00%	12	east	FP
Zhifeng Peng [66]	2016	98.57%	90.00%	24	east	FP
Zhiming Liu [67]	2021	90.91%	79.55%	12	east	FPPF
Zhiqiang Lin [68]	2020	90.67%	40.00%	12	east	FPP
Zilong Shi [69]	2022	94.12%	61.76%	40	east	FPP

### Quality assessment

**Fig. 3:**
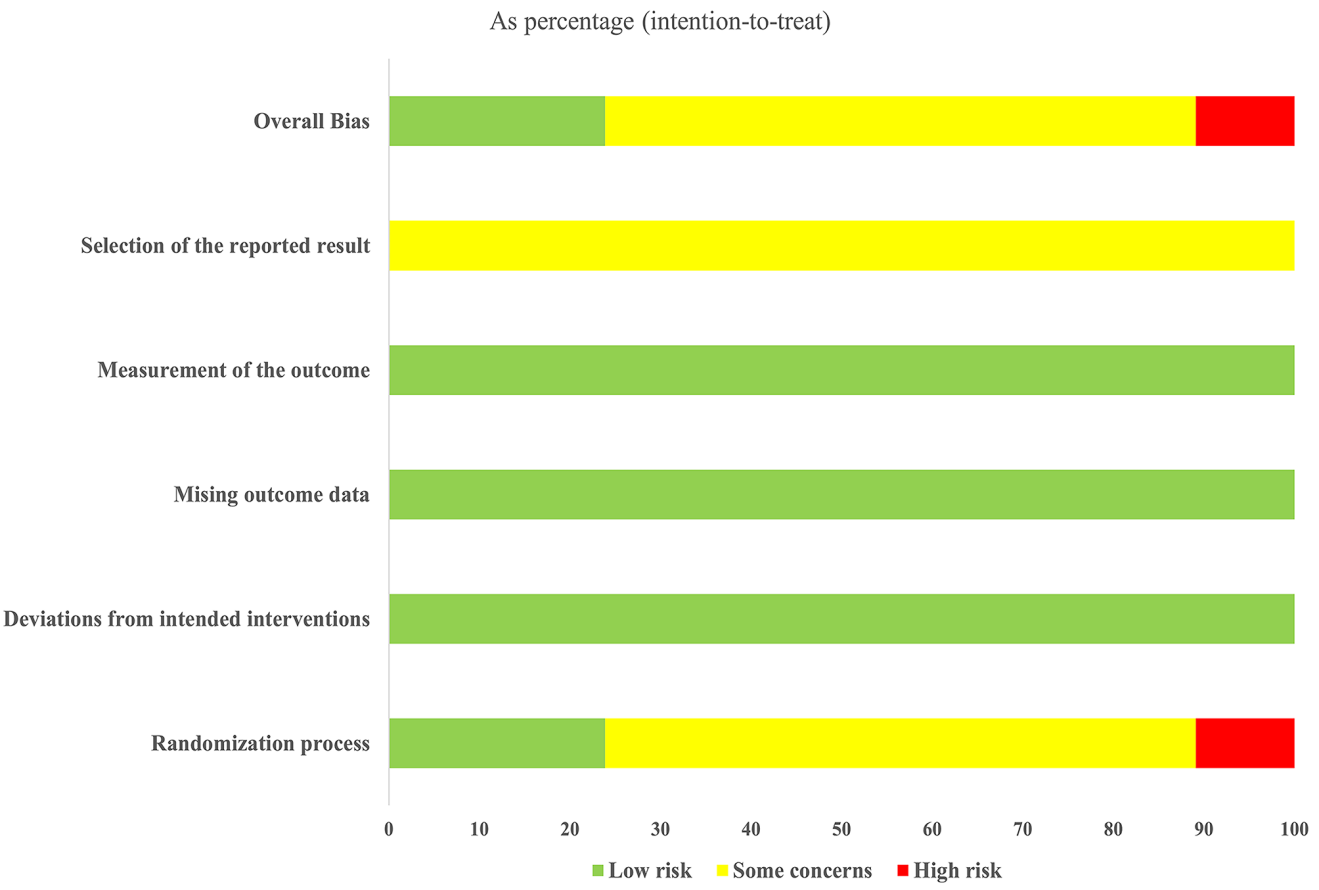
Risk of bias summary of the quality assessment

According to the results of the quality assessment in Figs. 3 and 23.9% of studies were assessed as “low-risk bias” (11 of 46 studies), 65.2% of studies were assessed as “some concerns” (30 of 46 studies), and 10.9% of studies were assessed as “high-risk bias” (5 of 46 studies).To be clear, all the studies had the subjects sign the informed consent form, so none of the studies were double-blinded, but the literature quality evaluators judged that the outcomes would not be affected by the lack of blinding, and selected “low-risk of bias”. No missing outcome data were identified and appropriate measurements of the outcome were used in all studies. Prior protocols were not found in all studies which were judged as “some concerns”.

### The control rate of hypertension

**Fig. 4:**
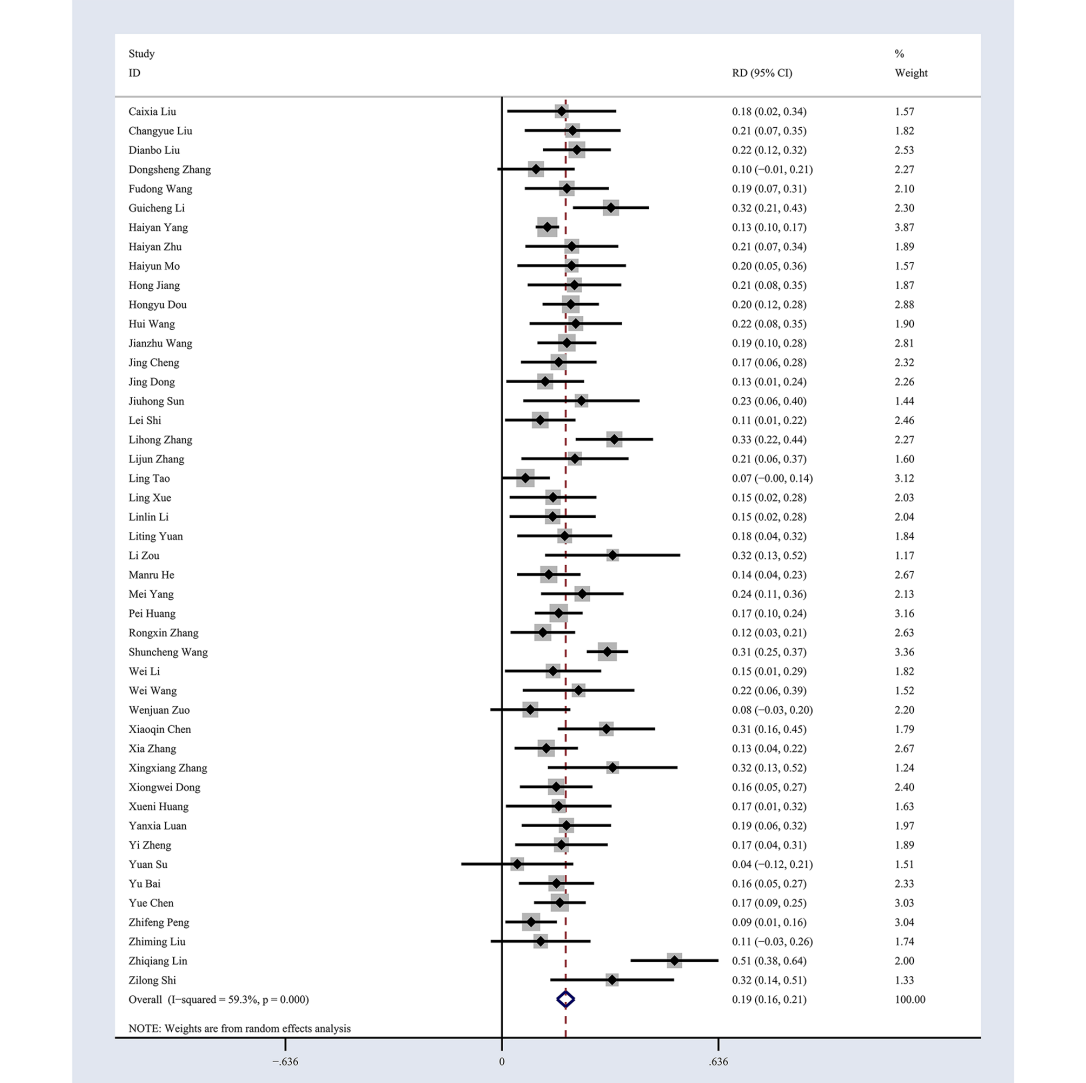
Forest plot for the control rate of hypertension under FPCS

According to the results of the meta-analysis, the FPCS can increase the control rate of hypertension by 19% (RD = 0.19, 95%CI: 0.16–0.21), and the difference was statistically significant (χ^2^ = 110.56, *P* < 0.001). As shown in Fig. 4, the 46 studies included have moderate heterogeneity ( I^2^ = 59.3%, *P* < 0.001), so the random effect model was used for the pooled analysis of effect size.

### Meta-regression

A meta-regression analysis was conducted to examine the effects of average age, experimental cycle, region, and intervention mode on the control rate of hypertension (Table 2). Average age (β = 0.28, *P* = 0.05) and intervention mode (β = 0.36, *P* < 0.001) have significant effects on the control rate.

**Table 2:** Meta-regression of the control rate of hypertension under FPCS

Covariate	β	Standard Error	95% Lower	95% Upper	Z	*p*-value
Average age	0.28	0.14	0.00	0.56	1.96	0.05
Experimental cycle	-0.15	0.17	-0.49	0.19	-0.88	0.38
Region	0.11	0.10	-0.07	0.29	1.24	0.22
Intervention mode	0.36	0.09	0.19	0.54	3.96	< 0.001

### Subgroup analysis

The average age and the mode of intervention are the sources of heterogeneity that impact the control rate of hypertension, as indicated by the meta-regression analysis results. To further investigate variations by age and by intervention model, subgroup analyses were carried out. The outcomes (Table 3) were as follows:

### The control rate of hypertension for different ages

The population under 65 is categorized as working-age, and the population 65 and older is classed as elderly, according to the National Bureau of Statistics of China’s age classification guidelines. There are 22 research that focus on the elderly population and 24 that deal with the working-age population. Table 2 illustrates that the elderly population has a greater control rate than people of working age (Supplementary Material 3).

### The control rate of hypertension by different intervention models

The three intervention modes were separated into FP, FPP, and FPPF. 25 of them employed the FP, 13 the FPP, and 8 the FPPF. According to the meta-analysis findings, FP, FPP, and FPPF had hypertension control rates of 90.1%, 94.4%, and 92.6%, respectively. Compared to the FP, the control rates of the FPP and FPPF were greater (Supplementary Material 3).

**Table 3:** Subgroup analysis of the control rate of hypertension under FPCS

Subgroup	Number of included studies	Heterogeneity Test		Model	The control rate of the experimental group (95CI%)
		I^2^/%	*P*-Value		
**Average age**					
44.00–65.00	24	58.6%	< 0.001	random	90.1% (86.9%, 93.3%)
65.01-75.00	22	60.7%	< 0.001	random	93.6% (92.1%, 95.2%)
**Intervention mode**					
FP	25	0%	0.745	random	90.1% (86.9%, 93.3%)
FPP	13	75%	< 0.001	random	94.4% (92.4%, 96.4%)
FPPF	8	74.3%	< 0.001	random	92.6% (89.3%, 96.0%)

### Publication bias

**Fig. 5:**
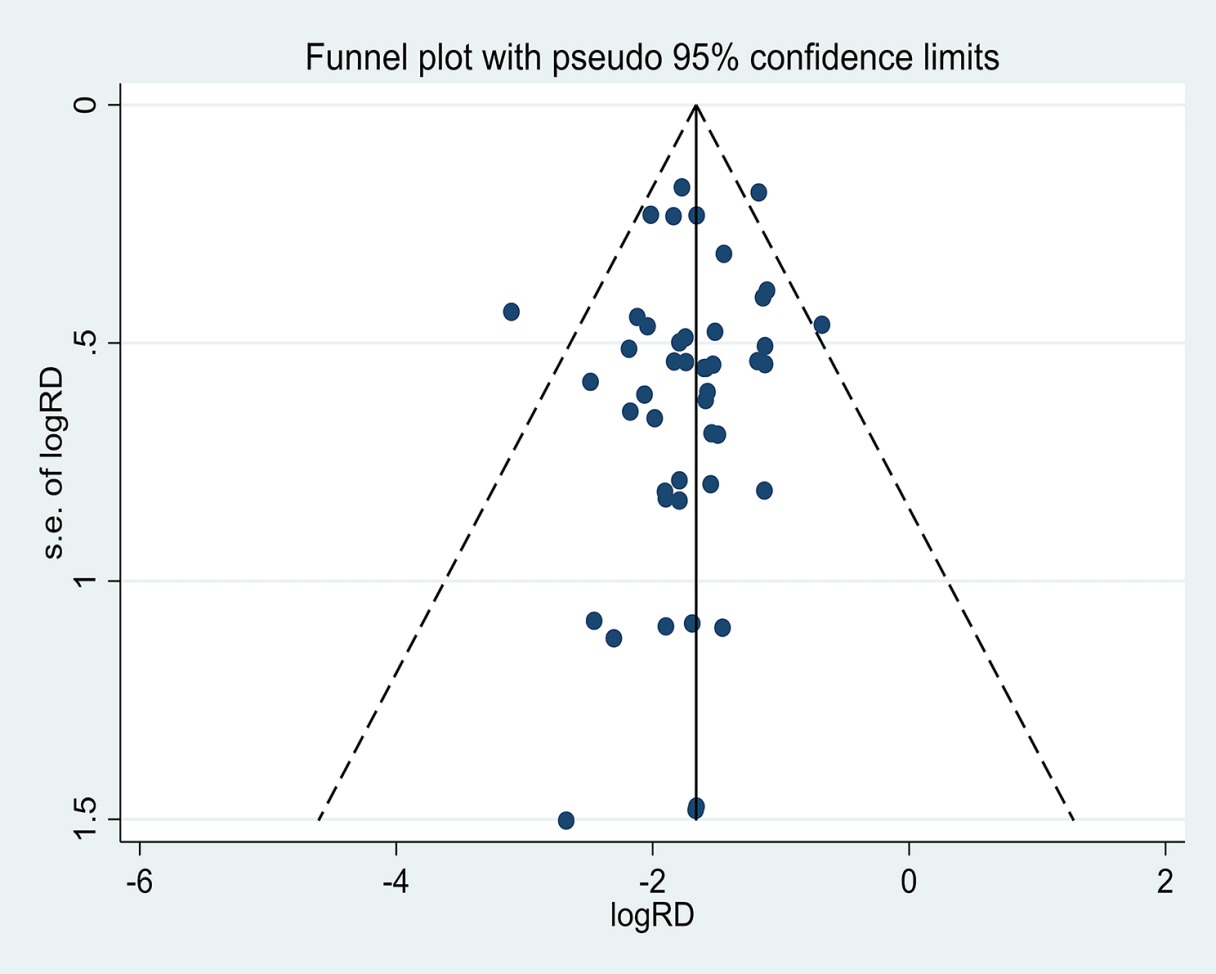
Funnel plot for the control rate of hypertension under FPCS

The funnel plot demonstrated that there was no evident publication bias and that the distribution of studies was fairly symmetrical. Additionally, there was no evident publication bias, as shown by the Egger test (*p* = 0.382 > 0.05) (Fig. 5).

### Sensitivity analysis

**Fig. 6:**
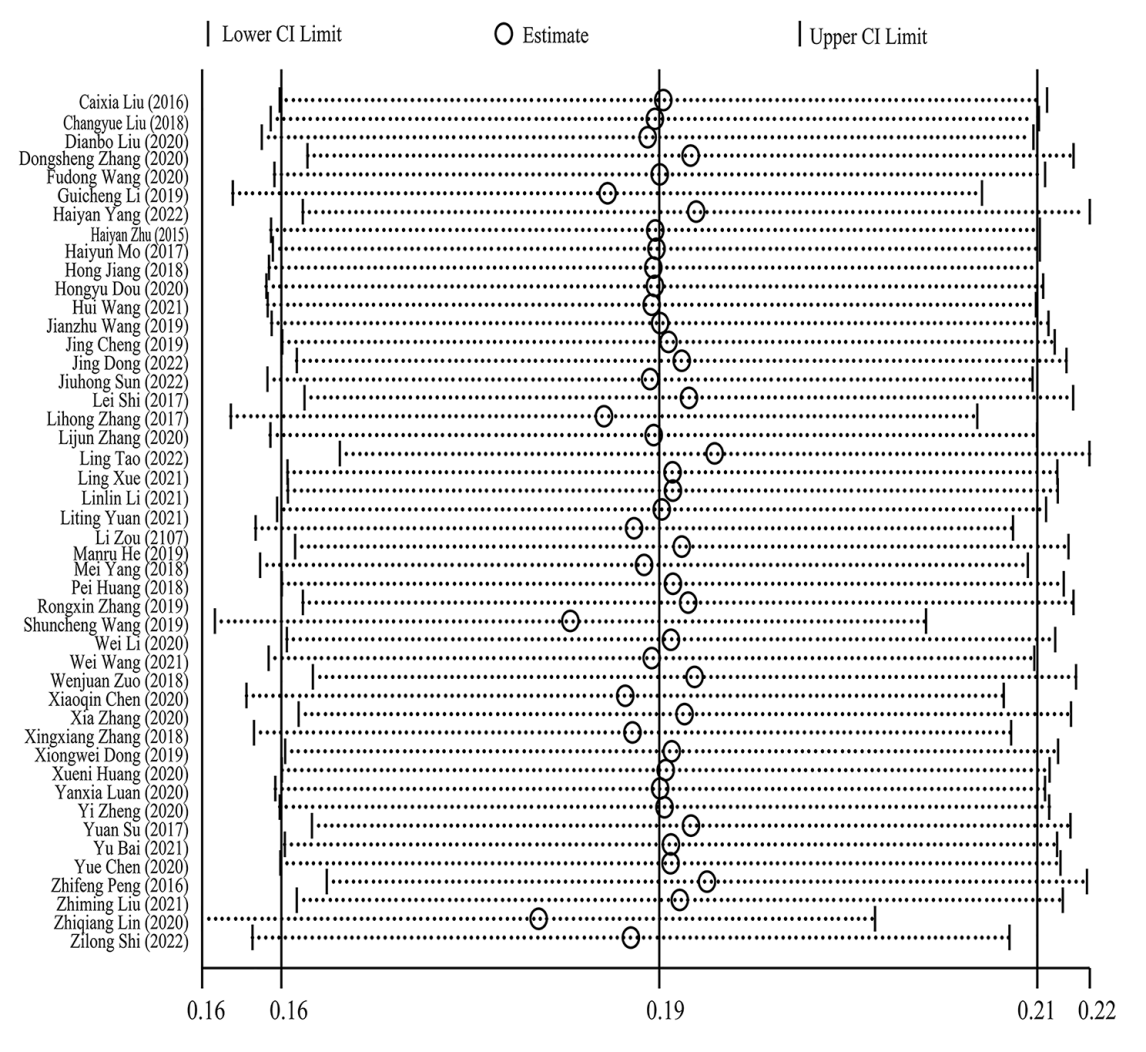
Sensitivity analysis by sequentially excluding each trial

Sensitivity analysis was performed using the one-by-one exclusion method to compare the pooled effect value with the results before the exclusion, and Fig. 6 shows that the conclusion was relatively stable.

## Discussion

### Summary of evidence

This study aims to evaluate the effect of family doctor signing services on the rate of hypertension control. A meta-analysis and systematic review revealed that FPCS could raise the rate of hypertension control by 19%. The average age and the intervention mode were found to be the sources of heterogeneity, according to the meta-regression results. Subgroup analysis was used to further explore the difference in average age and intervention mode. The results suggested that self-management and family support were key factors in the management of hypertension, that the elderly population had a better effect on hypertension control than the working-age population, and that the FPP and FPPF intervention had a better effect than FP.

### The influence of FPCS on the control rate of hypertension

Patients in the control group were intervened by routine community management or self-medication. The two key components of routine management in the community are phone follow-up and outpatient review. During the review, medical personnel will administer medicine, provide recommendations on diet and exercise, and provide health education based on the patient’s condition. The purpose of the phone follow-up is to check the blood pressure and schedule the review session. Self-medication intervention refers to hypertensive patients taking calcium antagonists, diuretics, and other antihypertensive drugs according to the doctor’s advice, and carrying out outpatient review after the treatment [70]. These two intervention models require excellent self-management skills, primarily relying on patients’ autonomy to seek care from grassroots medical institutions, concentrating on managing a particular disease condition rather than managing the patient [71]. However, in practice, patients’ capacity to self-manage their conditions is hampered by competing life circumstances. They frequently struggle to take their medications on time, have low self-management awareness, and are ignorant of the causes and treatments of hypertension, all of which contribute to inadequate illness control.

FPCS is a contractual cooperation between residents and nearby grassroots medical institutions. They are focused on enhancing patients’ quality of life, guaranteeing management continuity through routine patient health status monitoring, and offering advice on medication, diet, exercise, and other topics. It can effectively strengthen communication between doctors and patients, and improve the trust and compliance of patients. Furthermore, it will compensate for the shortcomings of traditional medical services, and solve the problem of poor effectiveness in patient self-management due to a lack of attention and supervision [72]. Nevertheless, the majority of current FPCS might overlook the effects of anxiety, depression, and other psychological issues on patients’ general health and drug compliance. It is urgent to provide targeted mental health services. Fortunately, there has been a progressive increase in awareness of the mental health issues that individuals with chronic illnesses face [73]. In short, the awareness, treatment, and control rates of hypertension in China have significantly improved recently, but they are still relatively low when compared to wealthy nations, and there is still much space for development in the management of hypertension [74].

### The influence of individual characteristics on the control rate of hypertension

The area has no discernible impact on the hypertension control effect, due to the study’s findings. According to a study, the eastern region’s contracted family doctors had a better effect on hypertension control than other regions [75]. This is because FPCS has a wealth of practical expertise and the eastern region has very accessible high-quality medical resources [76]. Yet, FPCS is implemented later in other places, and issues including a lack of family physicians and their caliber as well as an unscientific management system exist [77], which partially impedes the prevention and management of hypertension.

Compared with the working-age population, older people had a higher control rate for their hypertension, which is consistent with those of other studies [78, 79]. In addition to posing a longer medical history and a higher risk of complications like stroke, older patients also have higher levels of disease cognition [80], and their declining physical function means they will interact with the healthcare system more frequently and have more experience managing their condition, all of which are beneficial for blood pressure control [79]. Due to hectic work schedules, individuals of working age may not have the time to engage in health promotion activities like health education, which may result in a lack of initiative and compliance as well as a lack of information regarding the management of hypertension [81].

There is no obvious effect of the experimental cycle on the rate of hypertension control. The family physician’s contract service offers continuity features in theory. The beneficial influence on health-related behavior increases with the length of the contract [82]. A meta-analysis demonstrated that family physicians with contracts ranging from one to three years had a greater rate of hypertension control than those with contracts less than a year [75]. Nonetheless, Liang’s research revealed that, during the first three years of the intervention, the rate of hypertension management under general practitioners’ care rose, but in the fifth year, it fell in comparison to the third year [83]. It’s evident that the FPCS can, to a certain extent, improve the contracted objects’ health status, but this improvement may not last forever. Most studies have shown that the FPCS will continue to improve the patient’s compliance [84, 85], so this study speculates that the family doctor’s job burnout may be the reason for the drop in intervention intensity and service quality.

According to a systematic review on the topic, general practitioners experience moderate to high levels of job burnout globally [86]. This can have a detrimental effect on family physicians’ productivity, the quality of their services, and their patients’ health outcomes. The family practice system in China is currently improving gradually, but it also brings a work environment that increases pressure on family physicians with unreasonable workloads, arduous documentation burdens, etc. [68]. Family physicians may experience occupational burnout as a result of these reasons. As a result, it’s important to monitor job burnout to ensure the smooth development of family physician contracts.

### The influence of health behaviors on the control rate of hypertension

The effectiveness of two health behaviors, self-management and family support, in hypertension management was demonstrated in the current study. FPP emphasizes the importance of self-management in the prevention and control of hypertension, which is the lack of FP mode. Compared with group health education in FP, the advantage of MI is that it does not provide the same health education to all patients. Instead, according to the condition and self-management status of each patient, appropriate publicity and education methods are developed through discussion and consultation with patients, which pay more attention to the patient’s feelings and communication skills [87]. The relationship between knowledge, beliefs, and behavior is strengthened by the KAP model. Knowledge acquisition can help patients understand disease and treatment knowledge, which is the basis for correcting their thinking concepts; physicians play a vital role in changing patients’ attitudes and can help them overcome obstacles and build confidence; and finally, the formation of target behaviors based on knowledge and attitude can help patients adjust their physical and mental state, give play to their subjective initiative, and correct inappropriate behavior [88, 89]. A randomized controlled trial has shown that cognitive behavioral therapies increased patients’ self-efficacy levels and assisted them in improving metabolic control and health behaviors [90]. Additionally, a review found that cognitive behavioral therapy is an effective approach to enhancing medication adherence [91].

FPPF highlights the vital role of health workers in blood pressure control, which is neglected by FP. Many research outcomes have demonstrated that the support of family members was conducive to the improvement of the disease to a large extent, patients with stable families have a better state of health compared with patients from disrupted or isolated social circumstances [92]. The primary reasons are as follows: First, family health workers can provide health education and lifestyle guidance to patients as relatives, which makes it easier to gain the trust of patients and has higher compliance [93]; second, the participation of family members is a facilitator of positive self-management in patients, which can allow patients to feel more support and encouragement, and promote the improvement of self-efficacy [94]; third, timely family member care and communication can assist patients in lowering psychological stress, enhancing their self-confidence in their ability to fend off illness, maintaining a healthy mental state, and ultimately helping to normalize blood pressure [95].

### Suggestion

Based on the aforementioned findings and discussion, the present research makes the following recommendations:

First, it is suggested to further promote the application of FPCS in hypertension management, and at the same time, it is also necessary to pay more attention to the psychological state of patients with hypertension and provide personalized mental health services, to maximize the role of family doctors.

Additionally, other regions of China should learn from the experience of hypertension management in the eastern region to improve the service quality, and increasing research output should be emphasized to provide high-quality evidence to inform hypertension management.

Third, family physicians ought to tailor their medical care to each patient’s age-specific needs. For the working-age population who have less opportunity to participate in face-to-face services, it is suggested to use the blood pressure remote monitoring platform for real-time and whole process management of blood pressure.

Fourth, facilitate the performance appraisal system of family doctors, determine the remuneration through the actual workload and service output, and ensure the rationality of their income, to strengthen the service motivation of family physicians and reduce job burnout.

### Limitation

First, the majority of the studies included in this systematic review are from eastern China, while the studies in the central and western regions are fewer, and the representativeness of the results is limited. Second, in the included studies, scholars have several definitions of outcome indicators, which to some extent affected the heterogeneity between studies. Third, this study only included RCTs, and excluded Randomized self-control clinical studies, without verifying the difference in results caused by different experimental types.

## Conclusion

In conclusion, the family physician-contracted service has a high application value in the health management of hypertensive patients. The prevention and control of hypertension will depend on several elements, including average age, region, and intervention mode. To improve the quality of care provided by family physicians, efforts should be made to address the problems of uneven regional development, inadequate management of hypertension in the working-age population, and family physician job burnout. Patients and their families are encouraged to actively cooperate and work together to provide systematic, comprehensive, and targeted treatment services for patients, to promote the prognosis and quality of life of patients.

### Electronic supplementary material

Below is the link to the electronic supplementary material.

**Supplementary Material 1:** PRISMA 2020 Checklist

**Supplementary Material 2:** Search Strategies

**Supplementary Material 3:** Effect Sizes Forest Plots

## Data Availability

All data generated or analyzed during this study are included in this published article and its supplementary information files.

## Abbreviations

FPCS: Family physician-contracted service
RD: Risk difference
CI: Confidence interval
SBP: Systolic blood pressure
DBP: Diastolic blood pressure
CVD: Cardiovascular disease
CNKI: China National Knowledge Infrastructure
CQVIP: Chinese Scientific and Technological Journal Database
FP: “Family physician” model
FPP: “Family physician + Patient” model
FPPF: “Family physician + Patient + Family member” model
MI: Motivational Interviewing
KAP: “Knowledge – Attitude - Practice” model
