# Supplementary material for: Research on the control rate of hypertension under family physician-contracted service
Source: BMC Prim Care. 2024 Jan 31;25:47. doi: 10.1186/s12875-024-02280-0 (PMC10829220; doi:10.1186/s12875-024-02280-0)
Supplement: Supplementary file 1 — Supplementary Material 1: PRISMA 2020 Checklist [file 12875_2024_2280_MOESM1_ESM.docx]

**Supplement** **Table S1. PRISMA 2020 Checklist**

| **Section and Topic** | **Item #** | **Checklist item** | **Location where item is reported** |
| --- | --- | --- | --- |
| **TITLE** | | |  |
| Title | 1 | Identify the report as a systematic review. | Page 1 |
| **ABSTRACT** | | |  |
| Abstract | 2 | See the PRISMA 2020 for Abstracts checklist. | Pages 2-3 |
| **INTRODUCTION** | | |  |
| Rationale | 3 | Describe the rationale for the review in the context of existing knowledge. | Page 7-8 |
| Objectives | 4 | Provide an explicit statement of the objective(s) or question(s) the review addresses. | Page 6 |
| **METHODS** | | |  |
| Eligibility criteria | 5 | Specify the inclusion and exclusion criteria for the review and how studies were grouped for the syntheses. | Pages 9 |
| Information sources | 6 | Specify all databases, registers, websites, organisations, reference lists and other sources searched or consulted to identify studies. Specify the date when each source was last searched or consulted. | Pages 8-9 |
| Search strategy | 7 | Present the full search strategies for all databases, registers and websites, including any filters and limits used. | Supplement Table S2 |
| Selection process | 8 | Specify the methods used to decide whether a study met the inclusion criteria of the review, including how many reviewers screened each record and each report retrieved, whether they worked independently, and if applicable, details of automation tools used in the process. | Page 9-10 |
| Data collection process | 9 | Specify the methods used to collect data from reports, including how many reviewers collected data from each report, whether they worked independently, any processes for obtaining or confirming data from study investigators, and if applicable, details of automation tools used in the process. | Pages9-10 |
| Data items | 10a | List and define all outcomes for which data were sought. Specify whether all results that were compatible with each outcome domain in each study were sought (e.g. for all measures, time points, analyses), and if not, the methods used to decide which results to collect. | Pages9-10 |
|  | 10b | List and define all other variables for which data were sought (e.g. participant and intervention characteristics, funding sources). Describe any assumptions made about any missing or unclear information. | Page 9-10 |
| Study risk of bias assessment | 11 | Specify the methods used to assess risk of bias in the included studies, including details of the tool(s) used, how many reviewers assessed each study and whether they worked independently, and if applicable, details of automation tools used in the process. | Pages 10 |
| Effect measures | 12 | Specify for each outcome the effect measure(s) (e.g. risk ratio, mean difference) used in the synthesis or presentation of results. | Pages 11 |
| Synthesis methods | 13a | Describe the processes used to decide which studies were eligible for each synthesis (e.g. tabulating the study intervention characteristics and comparing against the planned groups for each synthesis (item #5)). | Pages 11 |
|  | 13b | Describe any methods required to prepare the data for presentation or synthesis, such as handling of missing summary statistics, or data conversions. | Pages 10-11 |
|  | 13c | Describe any methods used to tabulate or visually display results of individual studies and syntheses. | Pages 10-11 |
|  | 13d | Describe any methods used to synthesize results and provide a rationale for the choice(s). If meta-analysis was performed, describe the model(s), method(s) to identify the presence and extent of statistical heterogeneity, and software package(s) used. | Pages 10-11 |
|  | 13e | Describe any methods used to explore possible causes of heterogeneity among study results (e.g. subgroup analysis, meta-regression). | Pages 11 |
|  | 13f | Describe any sensitivity analyses conducted to assess robustness of the synthesized results. | Page 11 |
| Reporting bias assessment | 14 | Describe any methods used to assess risk of bias due to missing results in a synthesis (arising from reporting biases). | Page 11 |
| Certainty assessment | 15 | Describe any methods used to assess certainty (or confidence) in the body of evidence for an outcome. | N/A |
| **RESULTS** | | |  |
| Study selection | 16a | Describe the results of the search and selection process, from the number of records identified in the search to the number of studies included in the review, ideally using a flow diagram. | Pages 11,  Figure 2 |
|  | 16b | Cite studies that might appear to meet the inclusion criteria, but which were excluded, and explain why they were excluded. | Figure 2 |
| Study characteristics | 17 | Cite each included study and present its characteristics. | Pages 12-14,  Table 1, |
| Risk of bias in studies | 18 | Present assessments of risk of bias for each included study. | Pages 15-16 , Figure 3 |
| Results of individual studies | 19 | For all outcomes, present, for each study: (a) summary statistics for each group (where appropriate) and (b) an effect estimate and its precision (e.g. confidence/credible interval), ideally using structured tables or plots. | Page 16-17,  Figure 4 |
| Results of syntheses | 20a | For each synthesis, briefly summarise the characteristics and risk of bias among contributing studies. | Pages 18 , Figure 3 |
|  | 20b | Present results of all statistical syntheses conducted. If meta-analysis was done, present for each the summary estimate and its precision  (e.g. confidence/credible interval) and measures of statistical heterogeneity. If comparing groups, describe the direction of the effect. | Page 16-17 |
|  | 20c | Present results of all investigations of possible causes of heterogeneity among study results. | Page 17-18 |
|  | 20d | Present results of all sensitivity analyses conducted to assess the robustness of the synthesized results. | Page20,  Figure 6 |
| Reporting biases | 21 | Present assessments of risk of bias due to missing results (arising from reporting biases) for each synthesis assessed. | Page 19,  Figure 5 |
| Certainty of evidence | 22 | Present assessments of certainty (or confidence) in the body of evidence for each outcome assessed. | N/A |
| **DISCUSSION** | | |  |
| Discussion | 23a | Provide a general interpretation of the results in the context of other evidence. | Pages 20-21 |
|  | 23b | Discuss any limitations of the evidence included in the review. | Pages 27 |
|  | 23c | Discuss any limitations of the review processes used. | Pages 27 |
|  | 23d | Discuss implications of the results for practice, policy, and future research. | Pages 21-26 |
| **OTHER INFORMATION** | | |  |
| Registration and protocol | 24a | Provide registration information for the review, including register name and registration number, or state that the review was not registered. | N/A |
|  | 24b | Indicate where the review protocol can be accessed, or state that a protocol was not prepared. | N/A |
|  | 24c | Describe and explain any amendments to information provided at registration or in the protocol. | N/A |
| Support | 25 | Describe sources of financial or non-financial support for the review, and the role of the funders or sponsors in the review. | Pages 28 |
| Competing interests | 26 | Declare any competing interests of review authors. | Page 28 |
| Availability of data, code and other materials | 27 | Report which of the following are publicly available and where they can be found: template data collection forms; data extracted from included studies; data used for all analyses; analytic code; any other materials used in the review. | Page 28 |

**Supplement Table S2. Search Strategies**

| **Databases** | **Search terms** |
| --- | --- |
| **PubMed** | **#1** "family doctor"[All Fields] OR "family physician"[All Fields] OR "general practitioners"[All Fields] |
|  | **#2** "hypertension"[All Fields] OR "high blood pressure"[All Fields] |
|  | **#3** "China"[All Fields] OR "Chinese"[All Fields] |
|  | **#4** "control rate"[All Fields] OR "control ratio"[All Fields] OR "effective rate"[All Fields] |
|  | **#5** #1 AND #2 AND #3 AND #4 |
| **Web of Science** | **#1** "family doctor"[All Fields] OR "family physician"[All Fields] OR "general practitioners"[All Fields] |
|  | **#2** "hypertension"[All Fields] OR "high blood pressure"[All Fields] |
|  | **#3** "China"[All Fields] OR "Chinese"[All Fields] |
|  | **#4** "control rate"[All Fields] OR "control ratio"[All Fields] OR "effective rate"[All Fields] |
|  | **#5** #1 AND #2 AND #3 AND #4 |
| **Cochrane Library** | **#1** (family doctor):ti,ab,kw OR (family physician):ti,ab,kw  OR (General practitioners):ti,ab,kw |
|  | **#2** (hypertension):ti,ab,kw OR (high blood pressure):ti,ab,kw |
|  | **#3** (control rate):ti,ab,kw OR (control ratio):ti,ab,kw OR (effective rate):ti,ab,kw |
|  | **#4** (China):ti,ab,kw OR (Chinese):ti,ab,kw |
|  | **#5** #1 AND #2 AND #3 AND #4 |
| **CNKI** | (全文:家庭医生(精确))AND(全文:高血压+血压+慢性病(精确))AND(全文:控制率+达标率+有效率(精确))  **Type of literature:** journal article, Doctoral dissertation, Master dissertation |
| **Wanfang Data** | 全部:(家庭医生) and 全部:(高血压 or 血压 or 慢性病) and 全部:(控制率 or 达标率 or 有效率)  **Type of literature:** journal article, Doctoral dissertation, Master dissertation |
| **CQVIP** | (U=家庭医生) AND (U=高血压 OR 血压 OR 慢性病) AND (U=控制率 OR 达标率 OR 有效率)  **Type of literature:** journal article |

**Supplement Figure S1. Effect sizes forest plots for hypertension control rate under family physician-contracted services for different ages.**

**Supplement Figure S2. Effect sizes forest plots for hypertension control rate under family physician-contracted services by different intervention models**
