# Supplementary figures and images for: Research on the control rate of hypertension under family physician-contracted service
Source: BMC Prim Care. 2024 Jan 31;25:47. doi: 10.1186/s12875-024-02280-0 (PMC10829220; doi:10.1186/s12875-024-02280-0)

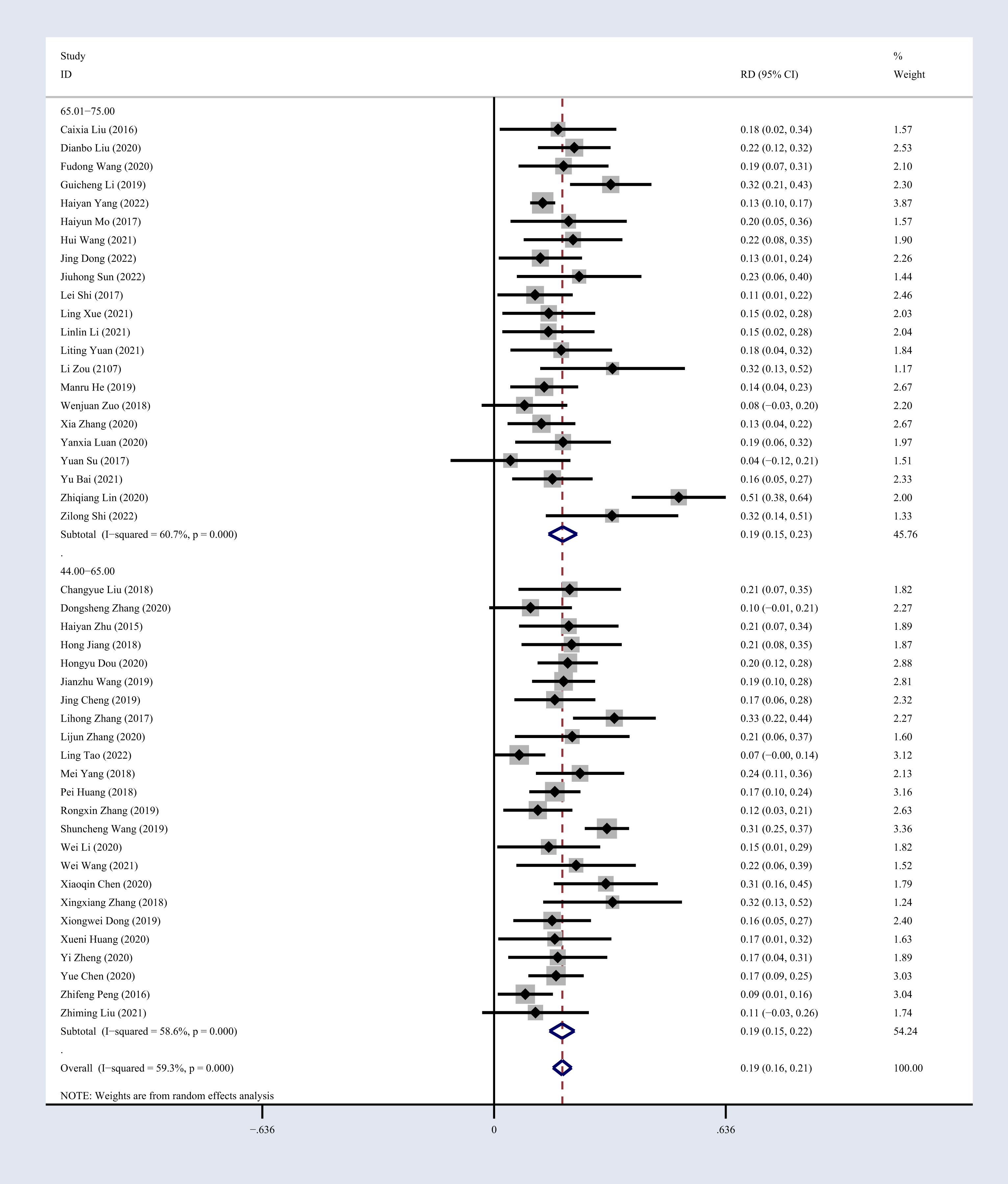

Supplement: Supplementary file 2 — Supplementary Material 2: Search Strategies [file 12875_2024_2280_MOESM2_ESM.tif]

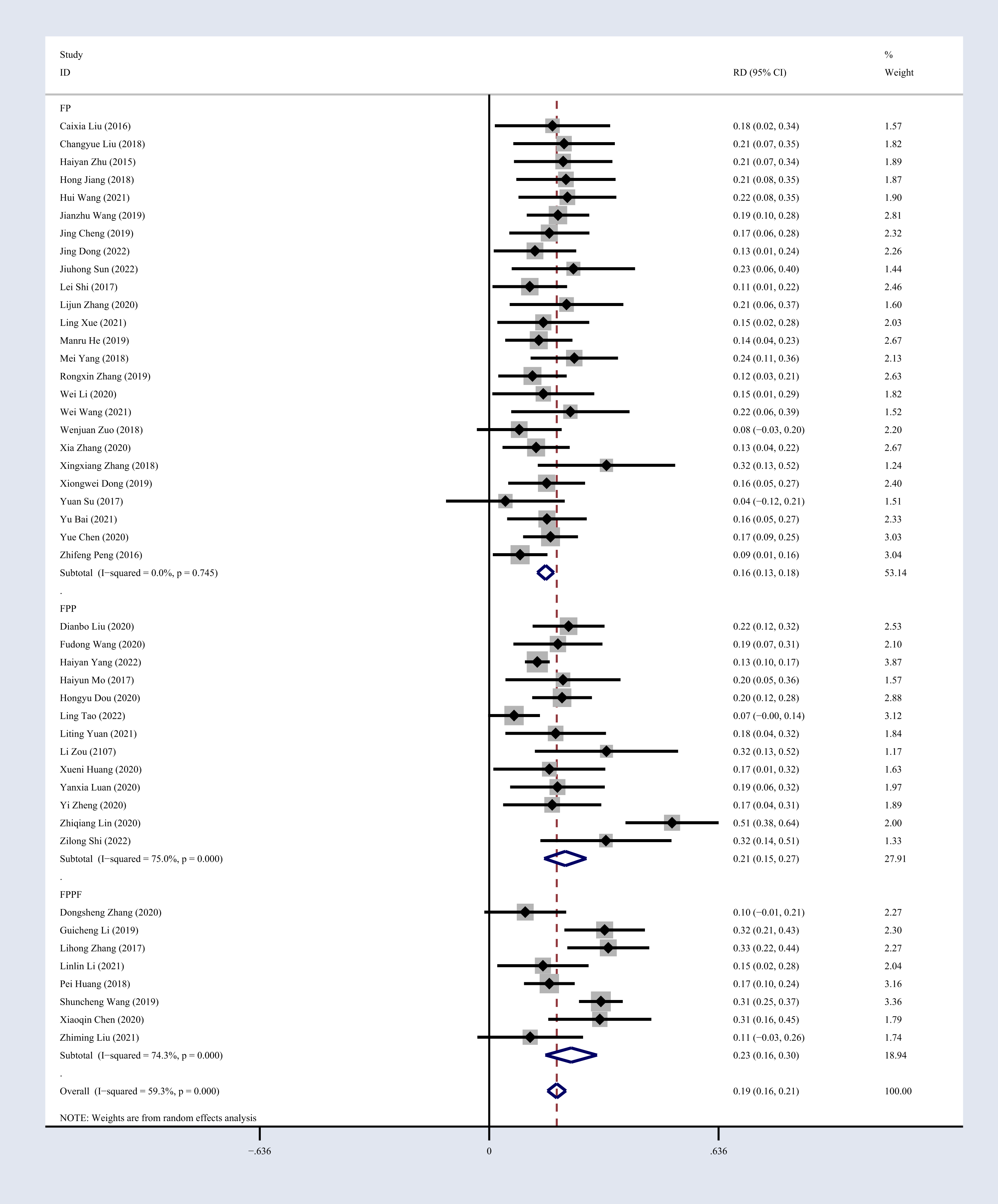

Supplement: Supplementary file 3 — Supplementary Material 3: Effect Sizes Forest Plots [file 12875_2024_2280_MOESM3_ESM.tif]
